# Severe phenotype of ATP6AP1‐CDG in two siblings with a novel mutation leading to a differential tissue‐specific ATP6AP1 protein pattern, cellular oxidative stress and hepatic copper accumulation

**DOI:** 10.1002/jimd.12237

**Published:** 2020-04-07

**Authors:** Nina Ondruskova, Tomas Honzik, Alzbeta Vondrackova, Viktor Stranecky, Marketa Tesarova, Jiri Zeman, Hana Hansikova

**Affiliations:** ^1^ Department of Pediatrics and Adolescent Medicine, First Faculty of Medicine Charles University and General University Hospital in Prague Prague Czech Republic

**Keywords:** ATP6AP1, congenital disorders of glycosylation, copper metabolism, glycosylation, metabolic disorder, oxidative stress

## Abstract

Congenital disorders of glycosylation (CDG) represent a wide range of >140 inherited metabolic diseases, continually expanding not only with regards to the number of newly identified causative genes, but also the heterogeneity of the clinical and molecular presentations within each subtype. The deficiency of ATP6AP1, an accessory subunit of the vacuolar H^+^‐ATPase, is a recently characterised N‐ and O‐glycosylation defect manifesting with immunodeficiency, hepatopathy and cognitive impairment. At the cellular level, the latest studies demonstrate a complex disturbance of metabolomics involving peroxisomal function and lipid homeostasis in the patients. Our study delineates a case of two severely affected siblings with a new hemizygous variant c.221T>C (p.L74P) in *ATP6AP1* gene, who both died due to liver failure before reaching 1 year of age. We bring novel pathobiochemical observations including the finding of increased reactive oxygen species in the cultured fibroblasts from the older boy, a striking copper accumulation in his liver, as well as describe the impact of the mutation on the protein in different organs, showing a tissue‐specific pattern of ATP6AP1 level and its posttranslational modification.

**Abbreviations**
ApoC‐III: apolipoprotein C‐III
BiP: binding‐immunoglobulin protein
CDG: congenital disorders of glycosylation
CHX: cycloheximide
DHE: dihydroethidium
Endo H: endoglycosidase H
ER: endoplasmic reticulum
ERAD: endoplasmic reticulum‐associated degradation
ICC: immunocytochemistry
ICP‐MS: inductively coupled plasma mass spectrometry
PNA: peanut agglutinin (lectin)
PNGase F: peptide: N‐glycosidase F
ROS: reactive oxygen species
TF: transferrin
tun: tunicamycin
VLCFA: very long chain fatty acids
WES: whole‐exome sequencing

## 1 INTRODUCTION

In 2016, a novel inherited metabolic disorder due to mutations in X‐linked *ATP6AP1* was identified by exome sequencing in 11 male patients from 6 families.^1^ Presenting with impaired protein N‐ and O‐glycosylation, it widened the known spectrum of congenital disorders of glycosylation (CDG), a heterogeneous group of diseases currently comprising more than 140 genetic defects. *ATP6AP1* codes for an accessory subunit of a vacuolar H^+^‐ATPase or V‐ATPase, which normally pumps cytosolic protons into the lumen of endocytic and secretory organelles and thus maintains the acidic pH to ensure their correct function (including Golgi glycosylation), as well as serves to acidify the extracellular space in specific cells.^2^ Up until now, mutations in four more genes encoding different V‐ATPase subunits (*ATP6V0A2*, *ATP6V1A*, *ATP6V1E1*, and *ATP6AP2*) have been found to underlie a glycosylation disorder in humans.^1^, ^3^, ^4^, ^5^

## 2 THE DIAGNOSIS OF ATP6AP1‐CDG IN OUR PATIENTS

**FIGURE 1:**
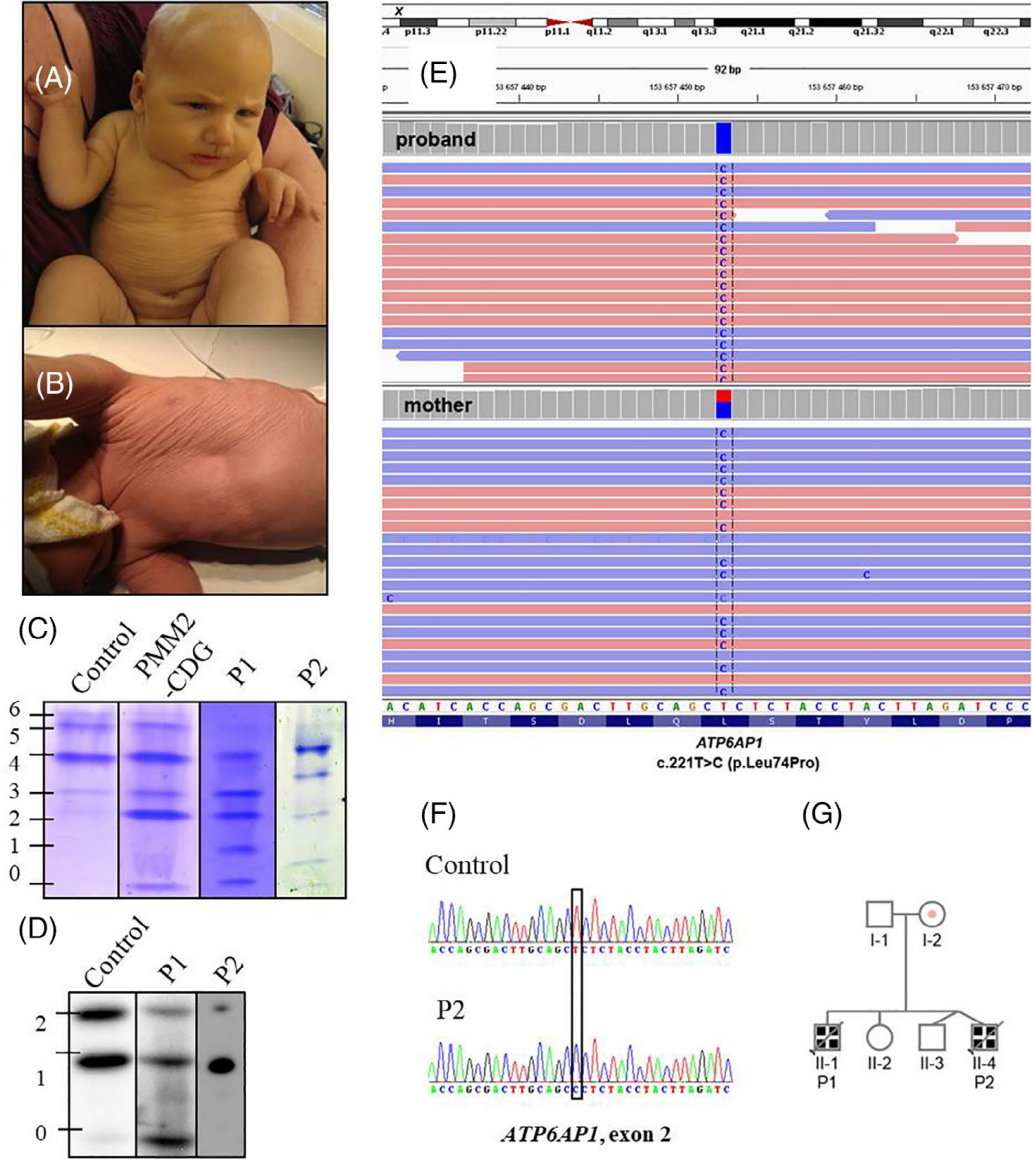
Diagnosis of ATP6AP1‐CDG in the siblings. The patients P1, A and P2, B both presented with persistent hyperbilirubinemia, hepatosplenomegaly, and cutis laxa, which in P2 disappeared after 6 months. Isoelectric focusing of serum transferrin, C and apolipoprotein C‐III, D, in the boys showed abnormal profiles consistent with a combined defect of N‐ and O‐glycosylation (numbers on the left indicate the number of sialic acid residues attached to the individual glycosylated forms of TF/ApoC‐III). Analysis by whole‐exome sequencing in P1, E, identified a hemizygous variation c.221T>C (p.L74P) in *ATP6AP1* gene, which was also heterozygously present in the mother. Sanger sequencing of *ATP6AP1* found the same mutation in P2, F. Distribution of this mutation in the affected family is shown in the pedigree chart, G

Here we outline a case of two male siblings (patient 1—P1, patient 2—P2; Figure 1A,B) from a non‐consanguineous Caucasian Czech family with a new pathogenic mutation in *ATP6AP1*, who presented with a very severe phenotype resulting in early death due to liver failure, while they showed no signs of immunodeficiency or neurologic involvement like the majority of the 13 previously described ATP6AP1‐CDG cases.^1^, ^6^, ^7^ For a comparison of selected clinical and laboratory data, see Table 1; the detailed description of our patients' clinical course is available in Supporting Information.

**TABLE 1:** Selected clinical and laboratory data in our patients compared to the described ATP6AP1‐CDG cases

Report	Jansen et al^1^											Witters et al^6^	Dimitrov et al^7^	our report		
Family	1			2	3		4		5		6	7	8	9		
Age	20 y	12 y	34 y	14 y	8 y	Died 4 y	23 y	18 y	Died 12 m	3 y	4 y	5 m	10 y	Died 3 m	Died 11 m	
cDNA mutation	c.1284G>A			c.431T>C	c.1036G>A		c.1036G>A		c.1036G>A		c.938A>G	c.649T>A	c.542T>G	c.221T>C		
Protein change	p.M428I			p.L144P	p.E346K		p.E346K		p.E346K		p.Y313C	p.Y217N	p.L181R	p.L74P		
Clinical findings																
Cutis laxa	NA	NA	NA	NA	NA	NA	NA	NA	NA	NA	NA	+	+	+	+	4/4
Abnormal liver biopsy	NA	NA	Normal	Slight steatosis	Fibrosis, steatosis, cirrhosis	Steatosis, cirrhosis	Micronodular cirrhosis	Micronodular cirrhosis	Fibrosis, steatosis, cirrhosis, cholestasis	NA	Fibrosis, steatosis, cirrhosis	Micronodular cirrhosis, steatosis	NA	Fibrosis, steatosis, cholestasis	Steatosis, cirrhosis, cholestasis	10/11
Infections	+	+	+	+	+	+	+	+	+	+	+	+	+	−	−	13/15
Hepatomegaly	+/−	−	−	−	+	+	+	+	+	+	+	+	+	+	+	12/15
Splenomegaly	−	−	−	−	+	+	+	+	+	−	−	+	+	+	+	9/15
Neonatal icterus	+	+	−	+	−	−	+	+	−	−	−	+	−	+	+	8/15
Neurologic symptoms	−	+/−	−	−	+	+	+	+	+	+	−	−	+/−	−	−	8/15
Laboratory findings																
Increased transaminases	+/−	+/−	+/−	+/−	+/−	+/−	+	+	+	+	+	+	+	+	+	15/15
Low serum copper and/or ceruloplasmin	+	+	NA	+	+	+	+	+	+	+	+/−	+	+	+	+	14/14
Hypogammaglobulinemia	+	+	+	+	+	+	+	+	+	+	+	−	+	−	−	12/15

Abbreviation: NA, (data) not available.

The family was screened for CDG using the examination of serum transferrin and apolipoprotein C‐III by isoelectric focusing, which revealed clear hypoglycosylated patterns in both affected boys, confirming a combined N‐ and O‐glycosylation defect (Figure 1C,D). P1 was referred for a genetic analysis using trio‐based whole‐exome sequencing, identifying a novel hemizygous mutation c.221T>C (p.L74P) in *ATP6AP1* gene, of which the mother was confirmed to be a carrier (Figure 1E). No variants were found in other glycosylation‐related genes that would be considered potentially disease‐causing. Later the same mutation was detected in P2 by Sanger sequencing (Figure 1F), while it was not present in his healthy twin, which supported the causality of this variant. The mutation was not found in GnomAD v2.1.1 nor 1000 Genomes Project databases, and it was not present in the control population from the region of the Czech Republic (>70 years, without severe diseases, 966 alleles). The in silico online tools MutationTaster, MutPred2, Panther, Pmut, Polyphen‐2, and PredictSNP predicted it to be either disease‐causing, deleterious or probably damaging (Table S1).

## 3 A COMPLEX DISTURBANCE OF ORGANELLE HOMEOSTASIS AND OXIDATIVE STRESS IN THE PATIENT'S CULTURED FIBROBLASTS

**FIGURE 2:**
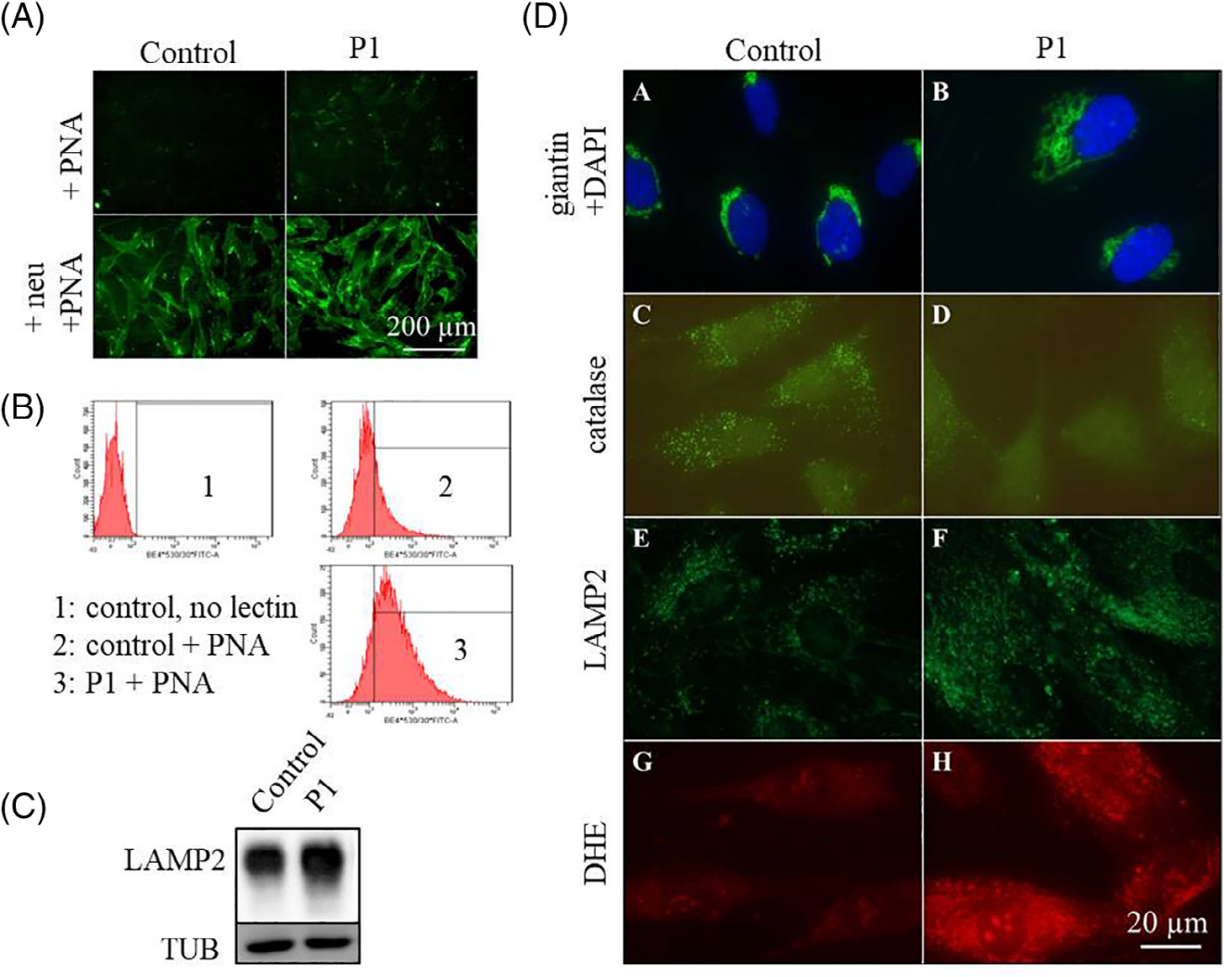
Altered cellular structure and ultrastructure found in P1's fibroblasts. Immunocytochemical (ICC) labelling of the fibroblasts with fluorescently tagged PNA lectin, A, showed an increased signal in P1 compared to the control (the upper part), suggesting a decreased sialylation of mucin type O‐glycoproteins; neuraminidase treatment prior to the staining (the bottom part) was used as a positive control. Quantification of the PNA binding was performed after the analysis of the labelled cells with flow cytometry, confirming the results discerned by ICC; histogram plots, B, show the fluorescent signal distribution in a healthy control vs P1 (*x*‐axis: fluorescence intensity; *y*‐axis: cell count). A complex defect in cellular glycosylation was corroborated by Western blot of LAMP2 (C), which revealed a smear with some additional bands in P1's sample corresponding to the underglycosylated forms of the protein. The structure of Golgi apparatus, D_A,B_, was visualised by ICC using anti‐giantin antibody, showing an altered morphology in the patient characterised by a higher ratio of cells with dilated Golgi (for multiple visual fields, see Figure S6); peroxisomal signal of catalase, D_C,D_, was decreased in P1; increased abundance of the lysosomal marker LAMP2, D_E,F_, was detected in P1, as well as elevated DHE signal reflecting a higher level of reactive oxygen species, D_G,H_. Quantification of ROS levels was performed using flow cytometry analysis after DHE staining, see Figure S7

First to characterise the glycosylation phenotype at the cellular level, P1's fibroblasts were assessed by immunocytochemistry (ICC) using PNA lectin (Figure 2A), which indicated overall hyposialylation of mucin type O‐glycoproteins. This result was corroborated by PNA lectin labelling followed by flow cytometry analysis, determining a ~3.2‐fold increase in the median value of P1's signal compared to the control's (Figure 2B). Defective glycosylation was also evinced by a Western blot analysis of the heavily N‐ and O‐glycosylated protein LAMP2 (Figure 2C). Next, Golgi apparatus was visualised by ICC labelling using anti‐giantin antibody, where P1's fibroblasts displayed a higher ratio of cells with altered morphology characterised by dilated Golgi structure (Figure 2D_A,B_). Peroxisomal perturbance as indicated by abnormal serum VLCFA levels in both patients was supported by ICC analysis of catalase, which showed a visibly decreased fluorescence signal localised to peroxisomes (Figure 2D_C,D_). The staining with LAMP2 antibody pointed to an increased abundance of lysosomes (Figure 2D_E,F_), concurring with autophagy disruption, which has been well described in ATP6AP2 defect.^5^ Furthermore, ATP6AP1 silencing in cancer cell lines was proven to lead to impaired autophagic flux.^8^ Intriguingly, immunofluorescent labelling with DHE probe revealed a striking increase in the level of cellular reactive oxygen species in P1 compared to the control (Figure 2D_G,H_). Our preliminary results have revealed variably elevated ROS also in other CDG subtypes, namely PMM2‐CDG, ALG8‐CDG, RFT1‐CDG, and SLC10A7‐CDG (Figure S1), showing that this is rather a universal phenomenon in CDG and not specific only to ATP6AP1 defect. We hypothesize that the elevated ROS could be linked to a chronic endoplasmic reticulum (ER) stress, as substantiated by the transcriptomic detection of activated unfolded protein response in fibroblasts from patients with different CDG subtypes (DPM1‐CDG, ALG6‐CDG, and ALG12‐CDG) in a study by Lecca et al,^9^ as well as by Dimitrov's^7^ observation of increased BiP expression as a marker of ER stress in fibroblasts from their ATP6AP1‐CDG patient.

## 4 A DRAMATIC COPPER ACCUMULATION DETECTED IN THE PATIENT'S LIVER

Another characteristic aspect of ATP6AP1‐CDG pathophysiology is the altered copper metabolism reflected by low serum copper and ceruloplasmin in the patients (see Table 1). We thus decided to examine the copper levels in P1's autopsy material including frontal cortex, heart, liver, skeletal muscle, and in the cultured fibroblasts by ICP‐MS, and saw a marked increase of copper content in his liver (286.7 μg/g dry weight; controls: 16.3‐44.4 μg/g dry weight); all the other tissues showed normal values (Table S2). No pathogenic variants in *ATP7A* or *ATP7B* have been found in P1 by WES. It is known that the copper transporting enzymes ATP7A/ATP7B reside mainly in the trans‐Golgi network, and presumably this specific environment, defined by lower pH, facilitates the release of Cu from these ATPases.^10^ At the same time, data show that altered pH homeostasis leads to aberrant Golgi morphology, causing mislocalization of Golgi‐resident proteins,^11^, ^12^, ^13^ likely including the copper pumps. The resulting impaired transfer of Cu onto the corresponding cuproenzymes, such as lysyl oxidase, might thus play a role in the aetiology of cutis laxa in the patients. Aberrant intracellular Cu transport could also be the underlying cause for our patient's hepatic copper retention, however the fact that it was limited to liver favours the assumption that the copper accumulation was secondary to his hepatic cholestasis, as supported by the report by Witters et al^6^ who noted a normalisation of ceruloplasmin in their infant ATP6AP1‐CDG patient after his cholestasis disappeared.

## 5 AN UNEXPECTED DIFFERENTIAL TISSUE‐SPECIFIC PATTERN OF ATP6AP1 AND ITS REDUCED STABILITY

**FIGURE 3:**
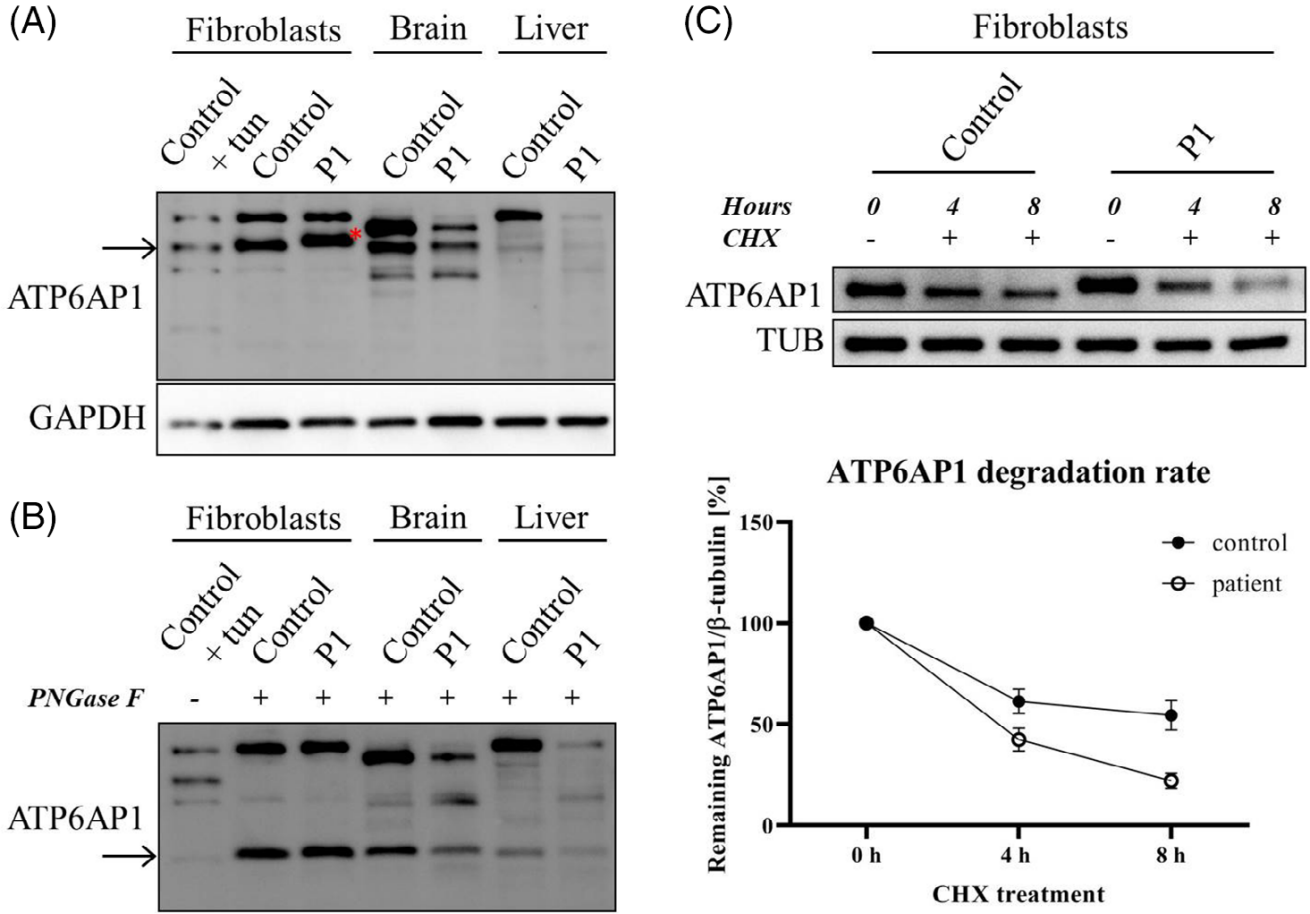
Protein analysis of ATP6AP1 in P1's tissues and his cultured fibroblasts. Western blot was performed in the whole cell lysates from fibroblasts and tissue homogenates. A, While ATP6AP1 (a band of ~ 62 kDa) was decreased in P1's frontal cortex and liver (to 29% and 42% of the control values, respectively), the signal in his fibroblasts was stronger than the control's (~1.7‐fold) and had a slower mobility (red asterisk). After PNGase F treatment, B, the band in all samples shifted to ~48 kDa, which corresponds to the mass of deglycosylated ATP6AP1, a small fraction of which is also present in cells cultured for 1 day with tunicamycin (tun; 5 μg/ml). Cycloheximide (CHX) chase assay, C, uncovered a reduced stability of the protein in the patient's fibroblasts (the degradation rate was compared with the relative amount of ATP6AP1 at the time point 0 hours set to 100%; mean values ± SEM are shown)

Western blot analysis of ATP6AP1 was carried out in the available tissues from P1, uncovering a significantly decreased amount of the full‐length 62‐kDa form in his frontal cortex and liver (Figure 3A). A surprisingly different pattern was seen in the patient's fibroblasts, where, in addition to a slight increase in the protein's amount, we noted its altered gel mobility—a shift to a higher molecular weight. The following enzymatic treatments of the cell lysates using PNGase F (Figure 3B) and Endo H (Figure S2) proved that this was due to a gain of its high‐mannose/hybrid type N‐glycosylation, implying a tissue‐specific regulation. The measurement of mRNA levels did not show any difference (Figure S3). Interestingly, despite the increased steady‐state level of ATP6AP1^L74P^, its stability was determined to be lower by a cycloheximide assay (Figure 3C). Twenty four‐hour transient transfection of the control's and patient's fibroblasts to produce FLAG‐tagged ATP6AP1^wt^ and ATP6AP1^L74P^ demonstrated the altered gel mobility of ATP6AP1^L74P^ to be mutation‐induced and independent on the genetic background, while further experiments indicated that the attachment of (an) extra N‐glycan(s) on the mutant protein is an early event in ATP6AP1 biosynthesis (Figures S4 and S5). Very interesting and relevant to our observation of ATP6AP1^L74P^ hyperglycosylation is the study by Rujano et al,^5^ who found increased STT3B‐dependent N‐glycosylation of the analysed ATP6AP2, another accessory subunit of V‐ATPase and an interacting partner of ATP6AP1, in fibroblasts from one of their ATP6AP2‐CDG subjects (p.L98S). Notably, the distinct N‐glycosylated band represented only a minor subpopulation of ATP6AP2^L98S^, as opposed to our finding where the whole fraction of ATP6AP1^L74P^ showed slower migration. They also demonstrated that this mutated protein, whose steady‐state level was found to be reduced in the patient's cells, was targeted for proteasomal degradation via ER‐associated degradation (ERAD) pathway. Considering the recognised role of STT3B in ER quality control, where it adds N‐glycans onto severely misfolded proteins to facilitate an alternative protein degradation pathway via N‐glycan‐dependent ERAD,^14^ it seems plausible that the gain of ATP6AP1 N‐glycosylation in our patient's fibroblasts might be a protective mechanism to remove the mutated protein failing to fold properly. The discrepancy in the protein pattern between the cultured fibroblasts and brain/liver samples might shed light on the differential organ involvement in the disease and poses an interesting question for future studies.

## 6 MATERIAL AND METHODS

Available in Supporting Information.

## CONFLICT OF INTEREST

The authors have no conflict of interest to declare.

## ETHICS STATEMENT

The study was performed in accordance with the Declaration of Helsinki and approved by the Ethics Committee of the General University Hospital in Prague.

## INFORMED CONSENT

All blood and tissue samples were analysed with informed consent from the parents of the patients.

## Supporting information


**Data S1.** Supporting information
