# Supplementary material for: Severe phenotype of ATP6AP1‐CDG in two siblings with a novel mutation leading to a differential tissue‐specific ATP6AP1 protein pattern, cellular oxidative stress and hepatic copper accumulation
Source: J Inherit Metab Dis. 2020 Apr 7;43(4):694–700. doi: 10.1002/jimd.12237 (PMC7383996; doi:10.1002/jimd.12237)
Supplement: Supplementary file 1 — Data S1. Supporting information [file JIMD-43-694-s001.docx]

**Supplement**

**Abbreviations**

ALT, alanine aminotransferase; aPTT, activated partial thromboplastin time; AST, aspartate aminotransferase; C22, docosanoic acid; C24, tetracosanoic acid; C26, hexacosanoic acid; CDG, congenital disorders of glycosylation; CHX, cycloheximide; DHE, dihydroethidium; Endo H, endoglycosidase H; GGT, gamma-glutamyltransferase; ICC, immunocytochemistry; ICP-MS, inductively coupled plasma mass spectrometry; iFBS, inactivated fetal bovine serum; INR, international normalised ratio; PFA, paraformaldehyde; PNA, peanut agglutinin (lectin); PNGase F, peptide:N-glycosidase F; ROS, reactive oxygen species; RT, room temperature; WES, whole-exome sequencing

**S1. Clinical course in the patients**

Patient 1 (P1) was born in term as the first child, with a birth weight 4010 g and length 52 cm. The early postnatal adaptation was uneventful, the only abnormality was hypospadia and icterus requiring continual phototherapy for three days. Cutis laxa predominantly on the chest and abdomen was noted (Fig. 1A) from the age of 3 weeks together with new jaundice, hepatomegaly, splenomegaly, cholestatic hepatopathy and increasing ascites with elevated levels of bilirubin (326 µmol/l; ref. val. < 29), direct bilirubin 13 µmol/l (ref. val. < 5), aminotransferases ALT 1.5 µkat/l (ref. val. < 0.73), AST 5.3 µkat/l (ref. val. < 1.21), GGT 6.0 µkat/l (ref. val. < 3), cholesterol 10 mmol/l (ref. val. < 4.3), alkaline phosphatase 22.0 µkat/l (ref. val. < 6.3), bile acids 246 µmol/l (ref. val. < 8) and coagulopathy (INR 1.79, ref. val. < 1.2; aPTT 70s, ref. val. < 42). Laboratory findings also showed decreased serum levels of copper (5.3 µmol/l; ref. range 13-23), low cerulopasmin (0.09 g/l; ref. range 0.2-0.6) and a borderline increase in very long chain fatty acids (C26 1.14 µkat/l, ref. val. < 0.5; C26/C22 0.063, ref. val. < 0.021; C24/C22 1.179, ref. val. < 1.09). The boy died at the age of 3 months due to progressive liver failure, and the autopsy confirmed intrahepatic cholestasis with liver fibrosis accompanied by significantly disorganized glandular parenchyma.

Patient 2 (P2), P1’s brother, was born from the third pregnancy as a dizygotic twin. Both his birth weight (3060 g) and length (48 cm) were normal. Transitory cutis laxa was apparent since birth to the age of 6 months (Fig. 1B). Phototherapy was administered on the 3rd day of his life due to increased bilirubin (265 µmol/l). He was discharged on the eighth day of his life, but was readmitted for phototherapy three times during the neonatal period due to sustained unconjugated hyperbilirubinemia with normal liver function tests. Mild elevation of GGT (3.8 µkat/l), AST (1.9 µkat/l), alkaline phosphatase 10.0 µkat/l (ref. val. < 1.21), cholesterol (7 mmol/l) and bile acids (122 µmol/l) were firstly documented at the age of 6 weeks. Increased concentration of bilirubin persisted (224 µmol/l; ref. val. < 4.3), and he was found to have markedly decreased ceruloplasmin level (0.09 g/l) and low level of antithrombin (40 %; ref. val. > 75); aPTT, INR, albumin level and creatine kinase activity were normal at that time. Hepatomegaly developed between the age of 4 and 6 weeks (liver from +2 cm to +4 cm below the costal margin). Similarly to his brother, the increase in very long chain fatty acids was observed (C26 0.61µkat/l, ref. val. < 0.5; C26/C22 0.05, ref. val. < 0.05; C24/C22 1.324, ref. val. < 1.09). He thrived well, had normal psychomotor development during the first 6 months of life, but the cholestatic liver disease progressed rapidly and eventually led to death due to liver failure at the age of 11 months. Hepatosplenomegaly (>10 cm below the costal margin), combined coagulopathy and cholestasis (bile acids > 400 µmol/l) were profound. The autopsy showed intrahepatic cholestasis with liver cirrhosis accompanied by diffuse nodular transformation with significant macrovesicular steatosis and ductular proliferation.

**S2. Material and methods**

**S2.1 Material**

Serum was separated from whole blood by centrifugation (630 g, 10 min, room temperature (RT)) and stored at -20 °C. Skin biopsy was performed in the patient 1 at the age of 2 months to obtain primary fibroblast lines; control dermal fibroblasts from male newborns were purchased commercially from ATTC (Manassas, Virginia). Tissue samples including frontal cortex, muscle, heart, liver from the patient 1 and 5 non-CDG controls were taken post-mortem within two hours, were immediately frozen and stored at -80 °C. The fibroblasts were cultured in DMEM medium (P04-04510, PAN Biotech) supplemented with 10 % inactivated fetal bovine serum (iFBS; SV30160.03, GE Healthcare Hyclone) and antibiotics (100x diluted XC-A4110, Biosera) at 37 °C in 5 % CO_2_ environment. Only fibroblasts with the passage number < 10 were used for the analyses; the cells were cultured to reach the confluence of ~ 80 % before the individual experiments were performed.

**S2.2 Methods**

**S2.2.1 Isoelectric focusing of transferrin and apolipoprotein C-III**

The methods were carried out as described previously [1, 2].

**S2.2.2 Genetic analysis**

Genomic DNA was extracted from peripheral blood using the standard procedure. Whole-exome sequencing (WES) of the trio patient 1 and his parents was performed using Illumina HiSeq 2000 system (Illumina) and SeqCap EZ Exome Enrichment  kit v3.0 (Roche NimbleGen). *ATP6AP1* gene (NG_052807.1, NM_001183.6) mutation identified by WES was confirmed by Sanger sequencing in the trio and the younger affected brother.

**S2.2.3 Cellular (ultra)structure and function study by immunocytochemistry and immunofluorescence**

The fibroblasts grown on coverslips were rinsed with PBS (BE17-517Q BioWhittaker, Lonza), fixed with 4 % paraformaldehyde (PFA; 19943 Affymetrix) for 10-15 min at 4 °C, permeabilized with 0.1 % Triton X-100 (T9284 Sigma-Aldrich) in PBS for 10-20 min at RT, blocked with 5-10 % iFBS in PBS for 1 hour (RT) and incubated with the corresponding primary antibody (for visualising Golgi: 1:200 giantin antibody, ab37266 Abcam; lysosomes: 1:100 LAMP2 antibody, sc-18822 Santa Cruz Biotechnology, Inc.; peroxisomes: 1:100 catalase antibody, A21987 Invitrogen; all diluted in blocking solution) at 4 °C overnight. The next day, the fluorescently-labelled secondary antibody (1:1000 anti-mouse IgG1-Alexa Fluor 488, A21121 Invitrogen; diluted in blocking solution) was added for 2 hours (RT), followed by nucleus staining with DAPI solution (D1306 Invitrogen, 10 µg/ml in PBS) for 10 min, RT (optional). In between all the steps and before image acqusition, the cells were thoroughly washed with PBS. Similar protocol was applied for peanut agglutinin (PNA) lectin staining, with the following modifications: for each tested cell line, the fibroblasts were cultured on two coverslips and prior to labelling, one of them was incubated with neuraminidase from *C. perfringens* (11585886001 Roche, 50 mU/0.25 ml medium) for 1 hour at 37 °C; the cells were then fixed with 1 % PFA in PBS (10 min, RT) and the solution containing Alexa Fluor 488-conjugated PNA lectin (L21408 Invitrogen, 5 µg/ml in PBS with 1 % iFBS) was added and left for 1 hour at 37 °C. For the *in vivo* detection of reactive oxygen species (ROS), the cells were incubated with dihydroethidium solution (DHE; D1168 Invitrogen; 5 µM in PBS) for 10 min at 37 °C. The signals were recorded by the epifluorescence microscope Nikon Diaphot 200 with the software Viewfinder (version 3.0.1, Pixera Corporation) either using automatic exposure mode (giantin, LAMP2) or at manually set exposure time (catalase, PNA, DHE); for each experiment, multiple images were taken and the representative ones were chosen for publication.

**S2.2.4 ATP6AP1 protein analyses using SDS-PAGE and Western blot, enzymatic treatment of the samples by PNGase F and Endo H, cycloheximide assay**

The fibroblasts for Western blot analysis were rinsed twice with ice-cold PBS and harvested by scraping into PBS; the cell suspensions were spun at 500 g for 5 min at 4 °C, and the pellets were collected and frozen at -80 °C. 5% (w/v) tissue homogenates were prepared from the muscle, brain and heart samples in KTEA buffer (150 mM KCl, 50 mM Tris-HCl pH 7.4, 2 mM EDTA, 0.2 μg/ml aprotinin) and from the liver samples in STEA buffer (250 mM sucrose, 20 mM Tris-HCl pH 7.4, 2 mM EDTA, 2 μg/ml aprotinin) at 4°C using Ultra-Turrax (IKA) and Potter–Elvehjem homogenizers (Bellco glass, Inc.); aliquots were stored at -80 °C. To prepare whole cell lysates, the fibroblast pellets or tissue homogenates were resuspended in RIPA buffer (50 mM Tris-HCl pH 7.4, 150 mM NaCl, 1 mM EDTA, 1 mM PMSF, 1 % Triton X-100, 1 % sodium deoxycholate, 0.1 % SDS) with 1 % protease inhibitor cocktail (P8340, Sigma-Aldrich), incubated on ice for 20 min with occasional vortexing, then spun (50 377 g, 20 min, 4 °C) and the supernatant was collected and stored at -80 °C until analysis. Protein concentration was determined by Lowry and BCA assays. The samples for tricine (and glycine, for LAMP2 detection) SDS-PAGE separation were prepared by mixing the whole cell lysates with 4x sample buffer (200 mM Tris-HCl pH 6.8, 48 % glycerol, 16 % SDS, 0.04 % bromophenol blue) and heating at 37°C for 30 min; 10-30 µg of total protein per well were loaded onto 10 % or 12 % gels, depending on the material analyzed and protein to be detected. After electroblotting the proteins onto PVDF membranes (Immobilon-P, Millipore) using semi-dry transfer method, the membranes were blocked with 5 % nonfat dry milk in TBS for 1 hour, followed by 2-hour incubation with the respective primary antibody diluted in 2 % milk in TBS with 0.1 % Tween (1:200 ATP6AP1 antibody sc-81886, Santa Cruz Biotechnology, Inc.; 1:1000 FLAG antibody F1804, Sigma-Aldrich; 1:5000 GAPDH antibody ab8245 Abcam; 1:500 LAMP2 antibody, sc-18822 Santa Cruz Biotechnology, Inc.; 1:1000 α-tubulin antibody 2125S, Cell Signaling Technology, Inc.), washed three times with 0.1 % TBST, and probed for 1 hour (RT) with the corresponding secondary antibody diluted in 2 % milk in 0.1 % TBST (1:4000 anti-mouse IgG-peroxidase A8924, Sigma-Aldrich or 1:5000 anti-rabbit IgG-peroxidase A0545, Sigma-Aldrich). After thorough washing with 0.1 % TBST and TBS, the chemiluminiscence signal developed with SuperSignal^TM^ West Femto Maximum Sensitivity Substrate (34096, Thermo Scientific) was detected using GBox Chemi Imaging System (Syngene), and the acquired bands were subsequently quantified densitometrically by Quantity One (Bio-Rad). PNGase F (EC 3.5.1.52) and Endo H (EC 3.2.1.96) treatments of the whole cell lysates (10-20 µg of total protein) were performed according to the manufacturer’s instructions (P0704S and P0702S, New England Biolabs). For cycloheximide assay, the fibroblasts were cultured with cycloheximide (CHX; C1988-1G, Sigma-Aldrich; 200 µg/ml) and harvested after 4 or 8 hours, prior to Western blot analysis as described above. The quantified values were statistically analyzed and the graph generated using GraphPad Prism 8.3.0.

**S2.2.5 Flow cytometry measurement of PNA lectin-labelled fibroblasts**

The cultured fibroblasts were rinsed twice with PBS and trypsinized; after the cells were detached, the trypsin was inactivated by adding 1 % iFBS in PBS, 5x10^5^ cells from each tested line were spun at 500 g, 5 min at RT and the supernatant discarded. After fixation with 4 % PFA in PBS for 20 min at RT, the fibroblasts were incubated with Alexa Fluor 488-conjugated PNA lectin (L21408 Invitrogen, 10 µg/ml in PBS with 1 % iFBS) for 1 hour on ice. In between the steps and after labelling, the cells were washed by adding 1 % iFBS in PBS, followed by centrifugation (500 g, 5 min, RT) and discarding the supernatant. Finally, each pellet was resuspended in 500 µl of 1 % iFBS in PBS and analyzed by BD FACS CANTO II flow cytometer (BD Biosciences) with the software FACSDiva Version 6.1.3. Forward scatter vs Side scatter gating was used to select a single-cell population, excluding debris or clumps, and the intensity of the green fluorescence was recorded in 20 000 events per sample.

**S2.2.6 Flow cytometry measurement of DHE stained fibroblasts**

The cultured fibroblasts were grown in medium with 0.1 % iFBS overnight, then rinsed twice with PBS and trypsinized to detach the cells. After trypsin inactivation with 2 % iFBS in PBS, 5x10^5^ cells per sample were spun at 500 g, 5 min at RT and the supernatant discarded. The pellet was resuspended in 500 µl of 10 µM DHE (dihydroethidium, D23107 Invitrogen) in HBSS (10-547F, Lonza/Bio-Whittaker), followed by 30-min incubation at 37 °C and measurement of the red fluorescence signal by flow cytometry (analogous to S2.2.5.). As a positive control (increased ROS production), we added 100 µM menadione (M5625, Sigma) to the control cells during the DHE incubation.

**S2.2.7 Determination of ATP6AP1 mRNA levels by qPCR**

The fibroblast pellets were resuspended in Tri Reagent (TR 118, Molecular Research Center, Inc.) and frozen at -80 °C prior to RNA extraction, which was subsequently performed using the manufacturer’s recommended protocol. cDNA was prepared from the corresponding RNA (1 µg per reaction), employing LunaScriptRT SuperMix (E3010S, New England Biolabs) and the programme: 1.) annealing: 25 °C, 2 min; 2.) cDNA synthesis: 55 °C, 10 min; 3.) heat inactivation: 95 °C, 1min. Quantitative real-time PCR was carried out with 50 ng of RNA reverse transcribed to cDNA using TaqMan-FAM ATP6AP1 probe (Hs00184593_m1, Thermo Fisher Scientific) and GAPDH probe (Hs99999905_m1, Thermo Fisher Scientific) in 7300 Real-Time PCR System (Applied Biosystems), with the following qPCR parameters: 1.) DNA polymerase activation: 95 °C, 10 min; 2.) amplification (40 cycles): 95 °C, 15 s; 60 °C, 1 min. The expression levels, calculated from Ct values, of *ATP6AP1* were normalized to *GAPDH*.

**S2.2.8 Copper content measurement by ICP-MS**

The measurement of copper content was performed using inductively coupled plasma mass spectrometry (ICP-MS). The sample was weighed into high-pressure Teflon (TFM) vessels and digested with a mixture of HNO_3_ and H_2_O_2_ in the microwave oven Milestone Ethos 1 (Milestone). After mineralization and evaporation of acids, the mineralizate was diluted with deionized water to the total volume of 10 ml. The determination of total copper concentration was performed using Elan DRC-e spectrometer (Perkin-Elmer SCIEX) equipped with a concentric glass nebulizer and quartz cyclonic spray chamber. The isotope ^63^Cu was used for quantification, and germanium ^74^Ge was used as an internal standard. The external calibration curve for copper was in the range of 0-50 µg copper/l, the concentration of the internal standard Ge was 10 µg/l. Blank samples were processed with the tested samples. The quality control was performed using the CRM NIST (The National Institute of Standards and Technology, USA) reference material samples, Oysters tissues 1566b with a certified value of 71,6 mg/kg. The recovery of the reference material was 91.6-93.5 %.

**S2.2.9** **ATP6AP1^wt^ and ATP6AP1^L74P^ overexpression in human fibroblasts**

The expression plasmid EX-T3068-Lv158 carrying human *ATP6AP1* cDNA reference sequence (NM_001183.6) with a C-terminal FLAG tag, referred to as “wild type”, was purchased from GeneCopoeia^TM^, and PCR site-directed mutagenesis was performed to produce the mutant plasmid with the variation c.221T>C (p.L74P) using the QuikChange II XL Site-Directed Mutagenesis Kit (200521, Agilent Technologies) and the designed oligomers 5’-CAGCGACTTGCAGCCCTCTACCTACTTAG-3’ and 5’-CTAAGTAGGTAGAGGGCTGCAAGTCGCTG-3’ from Generi Biotech. The plasmids (2000 ng/µl) were transiently transfected into control or patient fibroblasts by electroporation (1700 V, 20 ms, 1 pulse) using Neon^TM^ Transfection System (Thermo Fischer Scientific) and the manufacturer’s recommended protocol. 24 hours post transfection, the cells were either harvested or subjected to cycloheximide (200 µg/ml) treatment for 4 hours before harvesting.

**Table S1. *In silico* pathogenicity evaluation of the mutation c.221T>C in *ATP6AP1.***

| **Tool** | **Prediction** |
| --- | --- |
| Pmut | Disease |
| MutPred2 | Deleterious/Disease-associated |
| Panther | Probably damaging |
| PolyPhen-2 | Probably damaging |
| PredictSNP | Deleterious |
| CADD* score | 26.2 |

*Combined Annotation Dependent Depletion

**
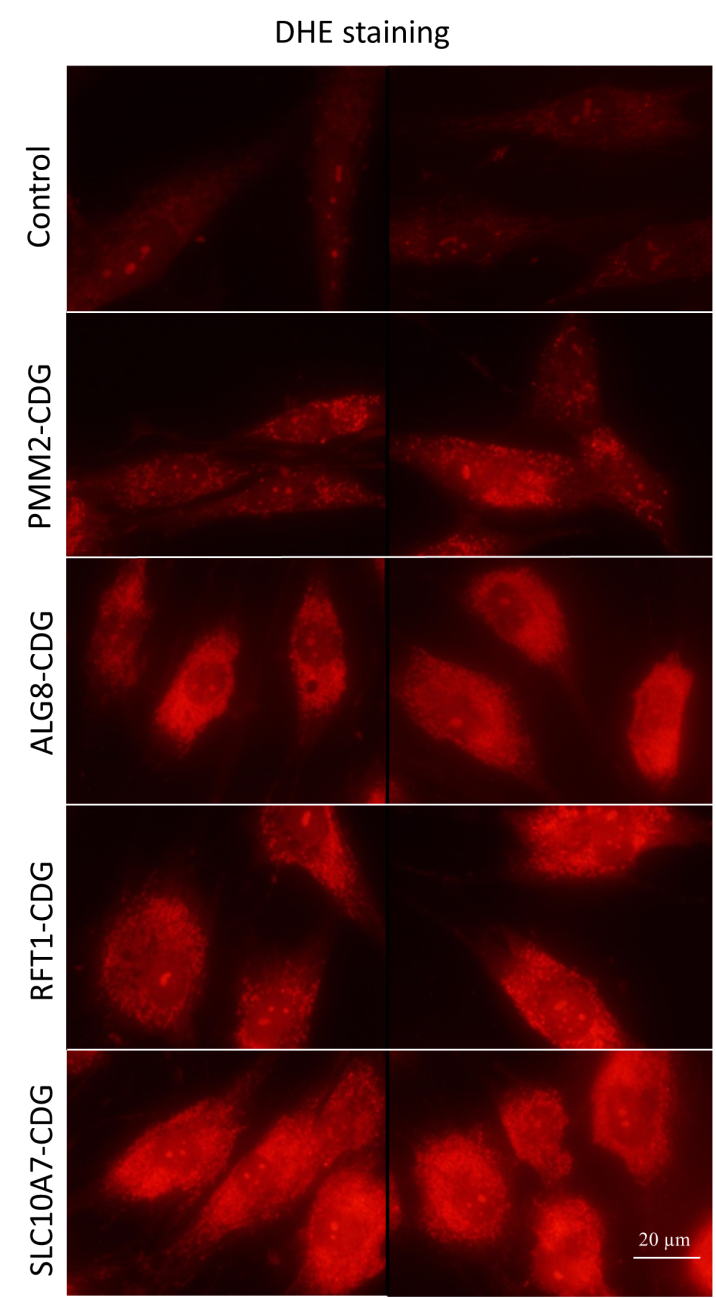
**

**Fig. S1. ROS detection in fibroblasts from patients with selected CDG subtypes.** DHE labelling to detect the level of intracellular reactive oxygen species (ROS) was performed in the cultured fibroblasts from a control and individuals with PMM2-CDG, ALG8-CDG, RFT1-CDG and SLC10A7-CDG (one patient from each type). Variably increased ROS compared to the control were observed in all the analyzed CDG subtypes.

**Table S2. Copper content measurement by ICP-MS in the available tissues from P1.**

| **Material** | **Copper [µg/g dry weight]* in controls** | **Copper [µg/g dry weight]* in P1** |
| --- | --- | --- |
| **Cultured fibroblasts** | 0.05 – 0.15 (n=3) | 0.11 |
| **Muscle** | 3.84 – 5.72 (n=4) | 4.94 |
| **Liver** | 16.3 – 44.4 (n=4) | **286.7** |
| **Heart** | 11.1 – 14.7 (n=3) | 13.4 |
| **Brain (frontal cortex)** | 9.61 – 21.9 (n=2) | 18.1 |

*detection limit: 0.02; limit of quantification: 0.06; uncertainty: ± 15%

**
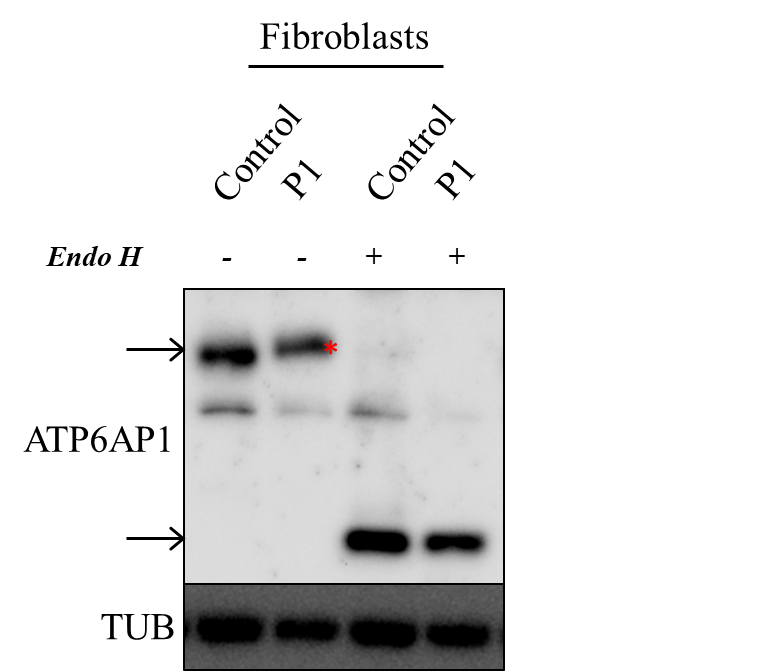
**

**Fig. S2. Endoglycosidase H treatment in control and P1’s fibroblasts.** Prior to Western blot analysis of ATP6AP1, the whole cell lysates (15 ug of total protein) from control and P1’s fibroblasts were treated with endoglycosidase H (Endo H). The mobility shift in P1’s sample - as indicated by the red asterisk – disappears after the treatment, arguing for the increased amount of high-mannose/hybrid N-glycans on untreated ATP6AP1^L74P^.

**Fig. S3. ATP6AP1 mRNA level in P1’s fibroblasts.** The expression level of *ATP6AP1* normalized to *GAPDH* in P1 was compared to three sex- and age-matched controls designated as c1-3 (c1 was used as the control cell line in immunofluorescence, immunocytochemistry, flow cytomtery and Western blot analyses in this study). X-axis shows the expression fold change of *ATP6AP1* in patient P1 relative to the control indicated on y-axis.

**
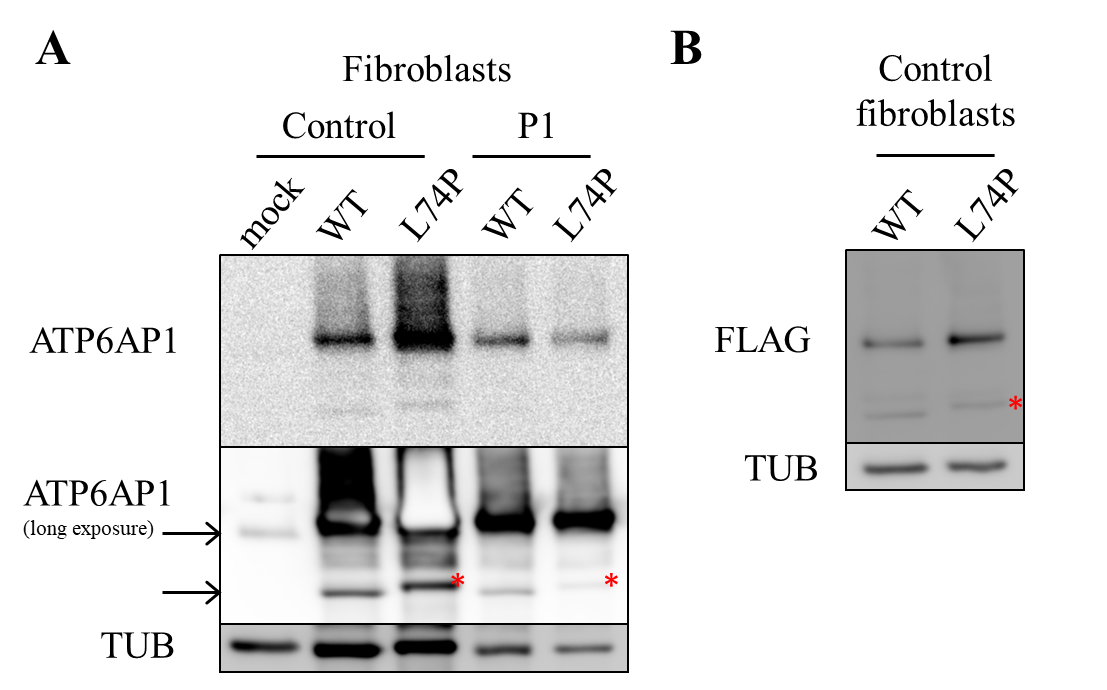
**

**Fig. S4. ATP6AP1^wt^ and ATP6AP1^L74P^ overexpression in control and P1’s fibroblasts.** Whole cell lysates (10 ug of total protein) from control and P1’s fibroblasts transiently transfected with FLAG-tagged ATP6AP1^wt^ or ATP6AP1^L74P^ were analyzed by Western blot using either ATP6AP1 (A) or FLAG (B) antibody. While the dominant full-length ATP6AP1^L74P^ did not exhibit a change in gel mobility, a shift was noted for a smaller band of ~ 50 kDa (red asterisk). The observed pattern was independent on the host cell line. We hypothesize that the lack of hyperglycosylation on the full-length form of the overexpressed ATP6AP1^L74P^ might result from the overwhelmed glycosylation capacity during protein translation, and that the band of ~ 50 kDa likely represents a yet unglycosylated nascent ATP6AP1, whose mutated variant is modified with (presumably) one N-glycan.

**
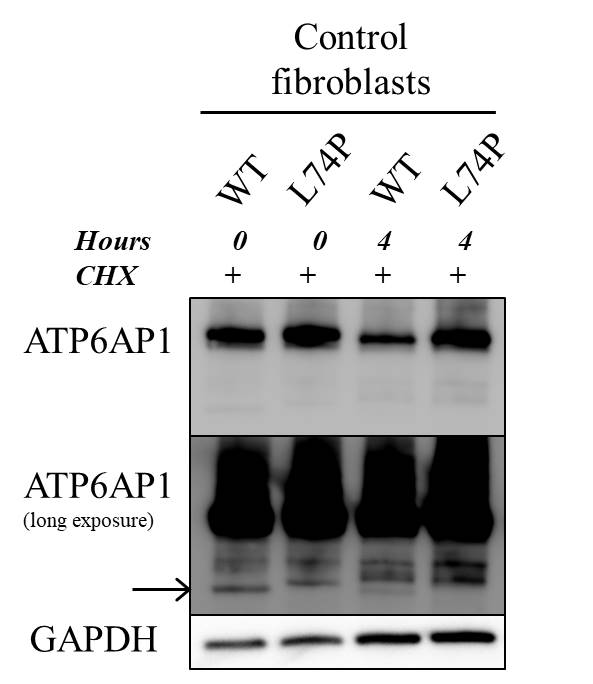
**

**Fig. S5. Cycloheximide treatment in cells overexpressing ATP6AP1^wt^ and ATP6AP1^L74P^.** 24 hours after transfecting control fibroblasts with FLAG-tagged ATP6AP1^wt^ or ATP6AP1^L74P^, the cells were incubated with cycloheximide (CHX; 200 µg/ml) for 4 hours and then harvested for immunodetection of ATP6AP1 by Western blot. The ~ 50 kDa band with differential gel mobility between ATP6AP1^wt^ and ATP6AP1^L74P^ decreases after the treatment, suggesting it is an intermediate product of ATP6AP1 biosynthesis.

**
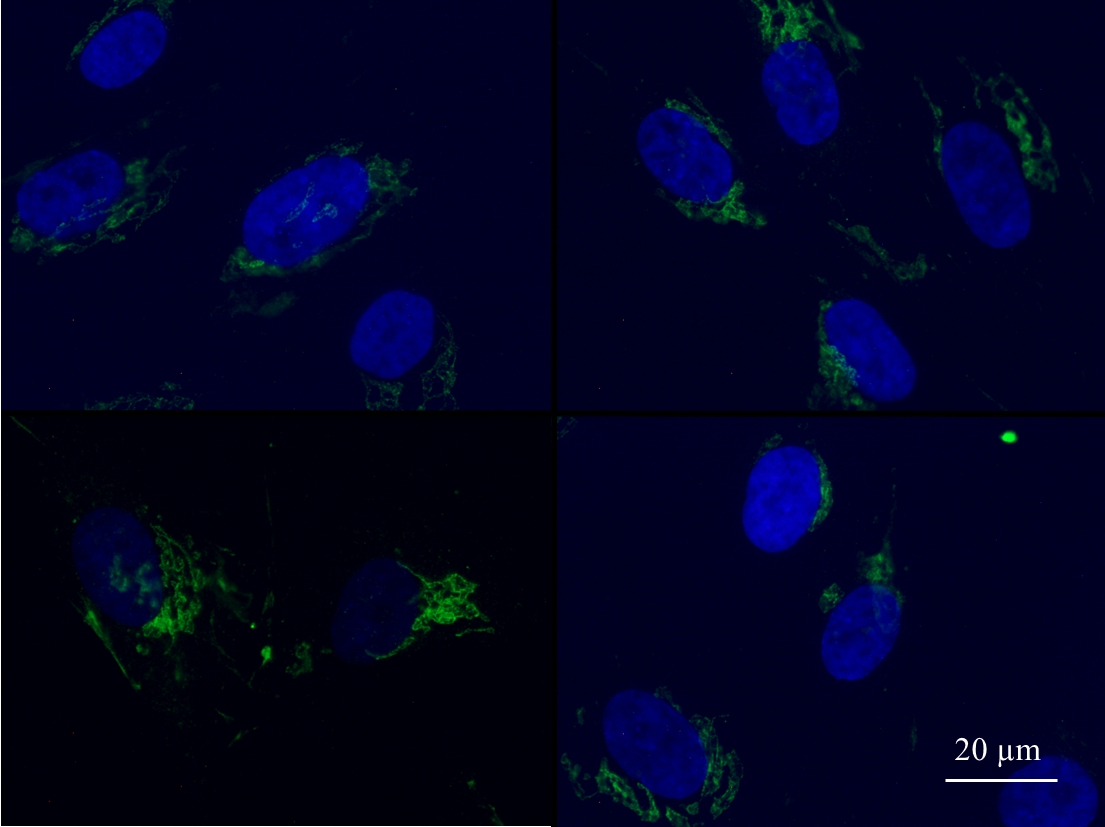
**

**Fig. S6. Altered Golgi apparatus morphology in P1’s fibroblasts.** ICC was performed using anti-giantin antibody (Golgi apparatus, green signal) and DAPI (nucleus, blue signal). Multiple visual fields of the patient’s fibroblasts are shown to demonstrate a higher ratio of cells with abnormal, dilated Golgi structure.

**
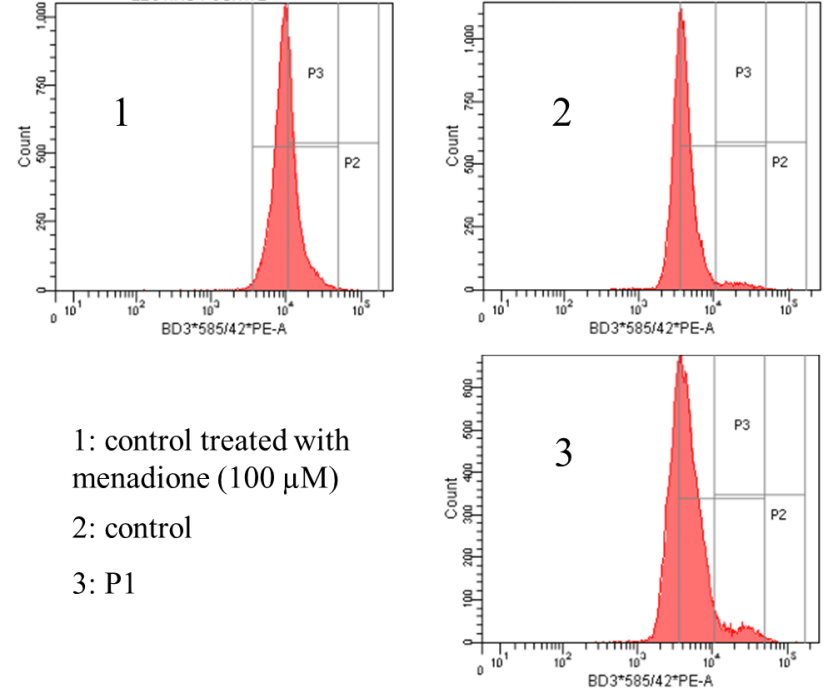
**

**Fig. S7. ROS detection by flow cytometry afer DHE staining in P1’s fibroblasts.** Quantification of the DHE staining (reflecting superoxide levels) was performed using flow cytometry, corroborating the observation from the immunofluorescence analysis (Fig. 2D_G,H_). Histogram plots show the fluorescent signal distribution in a positive control (1; a healthy control treated with 100 µM menadione, median: 9519), a healthy control (2, median: 3765) and P1 (3, median: 4160); x-axis: fluorescence intensity; y-axis: cell count.

References

[1] Wopereis S, Morava E, Grünewald S, Adamowicz M, Huijben KM, Lefeber DJ, et al. Patients with unsolved congenital disorders of glycosylation type II can be subdivided in six distinct biochemical groups. Glycobiology. 2005;15:1312-9.

[2] Ondrušková N, Honzík T, Kytnarová J, Matoulek M, Zeman J, Hansíková H. Isoelectric Focusing of Serum Apolipoprotein C-III as a Sensitive Screening Method for the Detection of O-glycosylation Disturbances. Prague Med Rep. 2015;116:73-86.
